# Effectiveness of a flow confirmation study of a central venous port of the upper arm versus the chest wall in patients with suspected system-related mechanical complications

**DOI:** 10.1186/s12957-022-02565-7

**Published:** 2022-03-22

**Authors:** Hiroyuki Tokue, Azusa Tokue, Yoshito Tsushima

**Affiliations:** Department of Diagnostic and Interventional Radiology, Gunma University Hospital, 3-39-22 Showa-machi, Maebashi, Gunma 371-8511 Japan

**Keywords:** Flow confirmation study, Central venous port, Subclavian vein, Brachial vein, System-related mechanical complications

## Abstract

**Background:**

If mechanical complications associated with a central venous port (CVP) system are suspected, evaluation with a flow confirmation study (FCS) using fluorescence fluoroscopy or digital subtraction angiography should be performed. Evaluations of mechanical complications related to CVP of the chest wall using FCS performed via the subclavian vein have been reported. However, the delayed complications of a CVP placed in the upper arm have not been sufficiently evaluated in a large population. We evaluated the effectiveness of FCS of CVPs implanted following percutaneous cannulation of the subclavian (chest wall group) or brachial (upper arm group) vein.

**Methods:**

A CVP was implanted in patients with advanced cancer requiring chemotherapy. FCS was performed if there were complaints suggestive of CVP dysfunction when initiating chemotherapy.

**Results:**

CVPs were placed in the brachial vein in 390 patients and in the subclavian vein in 800 patients. FCS was performed in 26/390 (6.7%) patients in the upper arm group and 40/800 (5.0%) patients in the chest wall group. The clinical characteristics of the patients were similar in both groups. The duration of CVP implantation until FCS was significantly shorter in the upper arm group (136 ± 96.6 vs. 284 ± 260, *p* = 0.022). After FCS, the incidence of CVP removal/reimplantation being deemed unnecessary was higher in the upper arm group (21/26 [80.8%] vs. 26/40 [65.0%], *p* = 0.27). In the upper arm group, no cases of catheter kinking or catheter-related injury were observed, and the incidence of temporary obstruction because of blood clots that could be continued using CVP was significantly higher than that in the chest wall group (10/26 [38.5%] vs. 4/40 [10.0%], *p* = 0.012).

**Conclusions:**

FCS was effective in evaluating CVP system-related mechanical complications and deciding whether removal and reimplantation were required in both groups.

## Background

Implantable central venous ports (CVPs) are widely used in patients with chronic illnesses in whom long-term access to the central veins for intravenous administration for chemotherapy, nutrient admixtures, blood products, antibiotic therapy, and blood sampling is required. Implantable CVPs are safe and easy to use; hence, they have become more widely used since their introduction in the 1980s [1]. However, an increasing number of mechanical complications related to CVP have been reported. This might be because of physicians being unaccustomed to using CVPs [2].

Complications of CVP systems are classified into periprocedural early and delayed complications. Early complications include pneumothorax, vascular injury, incorrect arterial puncture, ecchymoma, and pain. Delayed complications include local infection, catheter-related infection, venous thrombosis, and mechanical complications (catheter malposition, catheter occlusion, catheter fragmentation, and fibrin sheath formation). CVP system-related mechanical complications lead to system malfunction [3–5]; moreover, reimplantation may be required. If mechanical complications associated with the CVP system are suspected, evaluation with a flow confirmation study (FCS) using fluorescence fluoroscopy or digital subtraction angiography is recommended [2]. The unnecessary replacement may be avoided by properly assessing the mechanical complications using FCS.

Recently, the number of CVPs placed in the upper arm has increased [6]. However, the delayed complications of CVP placed in the upper arm have not been sufficiently evaluated in a large population.

In this study, we evaluated the effectiveness of FCSs of CVPs placed in the upper arm and chest wall. Furthermore, we compared the FCS results of suspected system-related mechanical complications between CVPs in the upper arm and those in the chest wall in patients receiving intravenous chemotherapy. Understanding the characteristics of the mechanical complications at the indwelling site of the CVP would prove useful.

## Methods

This study was approved by the ethics committee of Gunma University Hospital. We retrospectively analyzed the placement of 1211 CVPs in 1123 patients with advanced cancer who required chemotherapy. All data were obtained from medical record review alone. All patients were admitted to our hospital and underwent CVP placement between January 2013 and December 2020. We changed from chest wall CVP via subclavian venous implantation to upper arm CVP implantation in August 2018, because upper arm CVP implantation is considered safe and decreases the incidence of early complications related to the procedure [6].

All CVPs were single-lumen and open-end type catheters. A 5-Fr DewX (Terumo Clinical Supply, Tokyo, Japan) or a 5-Fr Anthron PU catheter (Toray Medical, Tokyo, Japan) was used for CVP implantation in the chest wall via the subclavian vein or in the upper arm via the brachial vein. Written informed consent was obtained from all patients prior to the procedure.

Implantation of CVP systems was performed by interventional radiologists in an interventional suite or operating room using ultrasonography (US) and fluoroscopic guidance under local anesthesia. Periprocedural antibiotic prophylaxis was administered to all patients. CVPs were implanted following percutaneous cannulation of the subclavian or brachial veins. Following intravenous injection of chemotherapy through a specific puncture needle (non-coring needles, Huber needles for ports), the CVP was flushed by a physician or nurse using a 10-mL syringe filled with 10 mL sterile saline. If the patients were required to remove the needle at home, they received guidance in a written manual from a nurse regarding the procedure to flush the CVP and needle removal. If the CVP was not used frequently, it was flushed using 10 mL sterile saline every 1–2 months at our hospital.

**Fig. 1:**
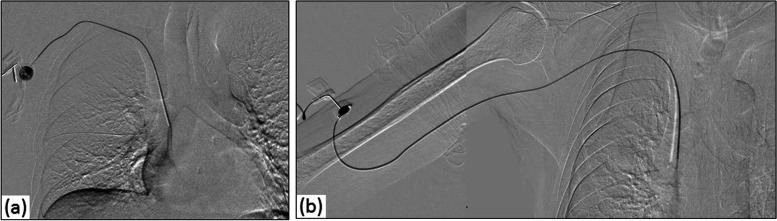
Normal flow confirmation study of central venous ports (CVPs). The catheter and port were in the expected location position but broken. Digital subtraction angiography with a manual injection of contrast material through the port revealed normal flow through the CVP system. No CVP system-related mechanical complications were observed. **a** Normal flow confirmation study of the CVP via the subclavian vein. **b** Normal flow confirmation study via the brachial vein

**Fig. 2:**
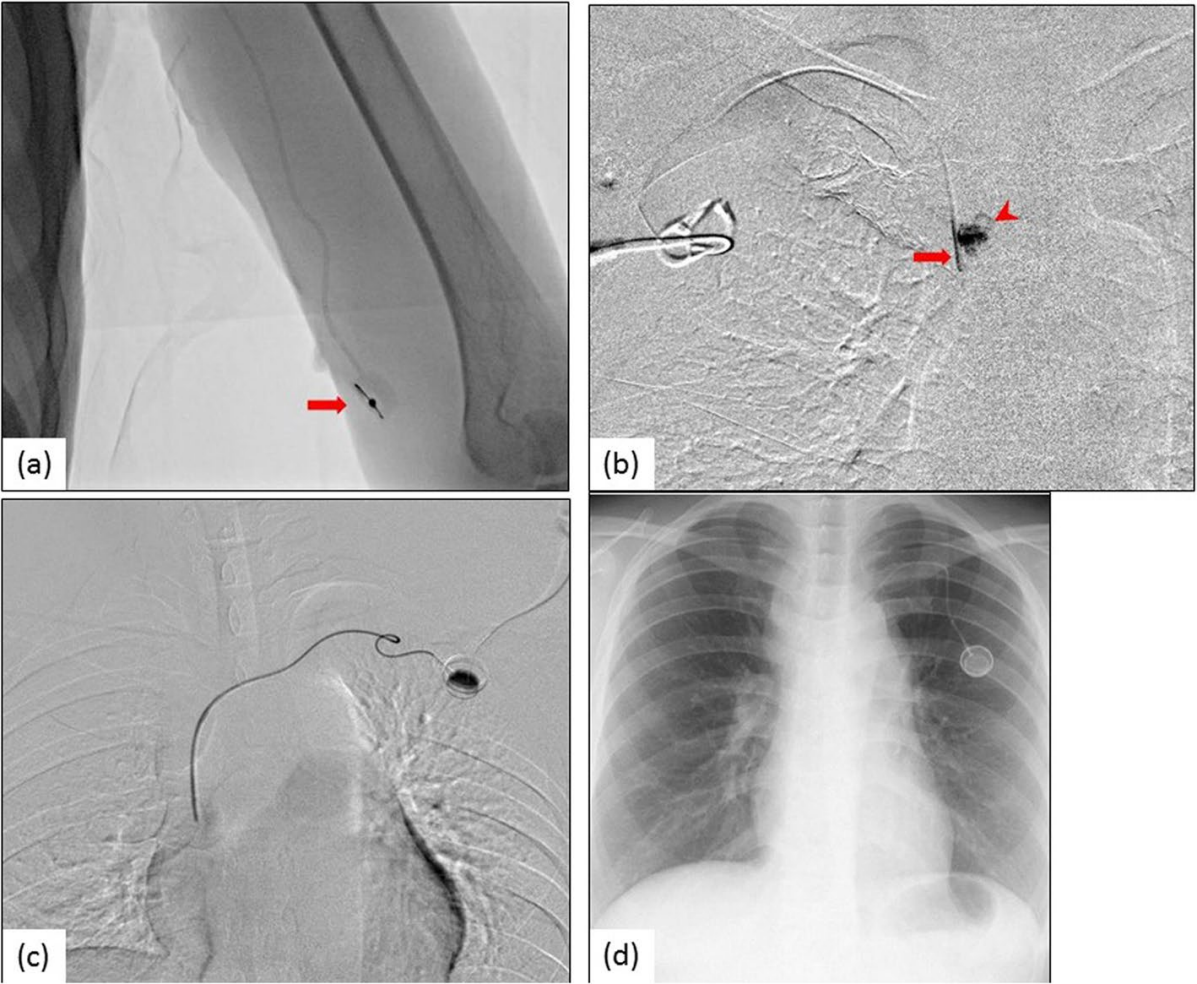
Abnormal flow confirmation study of central venous ports (CVPs). **a** A 57-year-old man with colon cancer complained that the specific puncture needle could not be inserted in the port. Fluoroscopy revealed irreversible port reversal (arrow). The CVP was removed. **b** A 73-year-old woman with gastric cancer complained of prolonged infusion time during chemotherapy. Chest fluoroscopy after the injection of contrast material revealed fibrin sheath formation around the tip of the catheter (arrow). Contrast material was not released from the tip of the catheter because of fibrin sheath formation (arrowhead). The CVP was removed. **c** A 65-year-old woman with colon cancer complained of prolonged infusion time during chemotherapy. Chest fluoroscopy while the patient was in the supine position revealed a secondary shift of the catheter course, but manual injection of contrast material through the port showed normal flow through the CVP system. **d** Chest X-ray revealed the course of the catheter was normal in the standing position. Therefore, chemotherapy was continued in a non-supine position

FCS was performed if there were complaints suggestive of CVP dysfunction when initiating chemotherapy (Figs. 1 and 2). However, if local infection or catheterization-related infections were suspected or if the physician judged it inappropriate, FCS was not performed, and the CVP was removed. FCS was performed in an angiographic suite (AXIOM Artis dTA; Siemens Medical Solutions, Erlangen, Germany) by interventional radiologists using fluoroscopy and digital subtraction angiography (DSA). First, the patient was placed in a supine position. Fluoroscopy was performed to check if the catheter and port were in the expected location and for breakages. We investigated whether the catheter position changes or a kink occurs because of the patient’s breathing, positioning, or arm movements. If the catheter’s kinking was released, depending on the situation, it was defined as a temporary kink, and if catheter kinking was not released by any means, it was defined as a permanent kink. Second, a specific puncture needle was inserted into the port to check whether saline could be manually injected without resistance, and then 10 mL of contrast medium was manually injected using fluoroscopy or DSA. If resistance was encountered upon injecting saline, the syringe was changed to 5 mL or 2.5 mL, and the procedure was attempted again with greater manual force. If manual injection with a non-resisting alternative syringe was possible and there were no abnormal findings in the FCS, the obstruction was considered to be temporary. If the injection was not possible even after changing the syringe, FCS was performed and the obstruction was considered permanent. Selective angiograms were obtained using a field of view that was closely collimated around the CVP system. The tube was placed under the table, and the input screen of the image intensifier was mostly in contact with the patient’s skin. The acquisition was performed with a frame rate of five frames per second to obtain multiple masks for subtraction with breath-holding. Angiography was performed in all patients undergoing FCS. Contrast medium injection was stopped when opacification of the CVP system and superior vena cava became apparent. Catheter or port injury was defined as leakage of contrast material through the catheter or port. Based on the FCS results, interventional radiologists decided whether the use of the CVP could be continued. After the FCS, the patients in whom CVP use was continued were followed up until death, until the CVP was no longer required and removed, or until April 2021.

Patients with a CVP placed via the subclavian vein between January 2013 and July 2018 were included in the chest wall group. Patients with a CVP placed via the brachial vein between August 2018 and December 2020 were included in the upper arm group.

The effectiveness of FCS of the CVP was evaluated based on whether mechanical complications related to the CVP system could be detected. The clinical characteristics reviewed included patient age, sex, reason for indwelling CVP, indwelling region of CVP, reason for FCS, duration of CVP implantation until FCS, abnormal FCS findings, and necessity of CVP reimplantation. We compared patient characteristics and FCS results between the upper arm and chest wall groups. In addition, we retrospectively examined the effectiveness of FCS.

Descriptive data are presented as mean ± standard deviation. The variables were compared between the two groups using Student’s *t*-test and the Mann-Whitney *U*-test or Fisher’s exact test. We used the SPSS v. 22 software (IBM Corp., Tokyo, Japan) for the statistical analyses, and *p* values < 0.05 were defined as statistically significant.

## Results

CVPs were inserted in the upper arm in 390 patients and in the chest wall in 800 patients. Twenty-one patients with CVP placed via the subclavian vein at the patient’s request between September 2018 and December 2020 were excluded. FCS was performed in 26/390 (6.7%) patients in the upper arm group and 40/800 (5.0%) patients in the chest wall group (repeated FCSs were not counted).

Table 1 shows the clinical characteristics of the patients in both groups. Age, sex, and type of cancer were similar between the groups. The incidence of FCS was slightly higher in the upper arm group; however, the difference was not significant.

**Table 1:** Clinical characteristics of patients who underwent a flow confirmation study

	Upper arm group	Chest wall group	*P*
*N*	26/390 (6.7%)	40/800 (5.0%)	0.28
Age (years)	64.6 ± 13.2	68.7 ± 16.3	0.24
Sex (man/woman)	12/14	23/17	0.65
Right/left side of CVP (*N*)	16/10	22/18	0.62
Type of cancer (*N*)			
Colorectal	15	22	0.14
Musculoskeletal	3	5	
Upper gastrointestinal	2	3	
Pancreas	2	1	
Breast	1	2	
Female pelvis	1	2	
Others	2	5	
Duration of CVP implantation until FCS (days)	136 ± 96.6	284 ± 260	*0.022
No abnormal findings in FCS (*n*)	9 (34.6%)	14 (35.0%)	0.59
After FCS, CVP removal/reimplantation was judged unnecessary (*n*)	21 (80.8%)	26 (65.0%)	0.27

*FCS* Flow confirmation study, *CVP* Central venous port

*Statistically significant

The duration of CVP implantation (days) until FCS was significantly shorter in the arm group (136 ± 96.6 vs. 284 ± 260, *p* = 0.022).

The incidence of no abnormal findings in the FCS was similar between the groups. After FCS, the incidence of CVP where removal/reimplantation was deemed unnecessary was higher in the arm group; however, there was no significant difference (21/26 [80.8%] vs. 26/40 [65.0%], *p* = 0.27).

Table 2 shows the complaints suggestive of CVP dysfunction and FCS outcomes. In the upper arm group, there were no cases of catheter kinking or catheter-related injury, and the incidence of temporary obstruction due to blood clots was higher than that in the chest wall group (10/26 [38.5%] vs. 4/40 [10.0%], *p* = 0.012). In the arm group, 17/21 patients continued using the CVP system following FCS without any system malfunction until death or removal of CVP at the end of therapy, after an average of 272 ± 85 days. Temporary obstruction occurred in four patients following the initial FCS, after an average of 42 ± 34 days. There were no patients who underwent a second FCS in the arm group. In the chest wall group, 21/26 patients continued using the CVP system following FCS without any system malfunction until death, and CVP removal at the end of therapy after 232 ± 132 days. There were four patients who repeatedly experienced mechanical complications and required a second FCS in the chest group. Temporary obstruction occurred in two patients following the first FCS after 32 and 50 days. CVP removal because of fibrin sheath formation occurred in two patients following the first FCS after 70 and 82 days. Death because of progression of the primary disease occurred in one patient following the first FCS after 111 days.

**Table 2:** Complaint suggestive of central venous port dysfunction and flow confirmation study outcomes

	Upper arm group (*N* = 26)	Chest wall group (*N* = 40)	*P*
Prolonged infusion time	12 (46.2%)	18 (45.0%)	0.56
▪ Temporary obstruction (relieved by strong flushing)	6	2	
▪ Fibrin sheath formation	2	3	
▪ Temporary catheter kinking	0	5	
▪ Permanent catheter kinking	0	2	
▪ No abnormal findings	4	6	
Inability or resistance to inject saline	7 (26.9%)	10 (25.0%)	0.54
▪ Temporary obstruction (relieved by strong flushing)	4	2	
▪ Fibrin sheath formation	1	1	
▪ Permanent obstruction	1	3	
▪ Temporary catheter kinking	0	2	
▪ Permanent catheter kinking	0	1	
▪ No abnormal findings	1	1	
Subcutaneous extravasation of chemotherapy drug	2 (7.7%)	4 (10.0%)	0.56
▪ Catheter-related injury	0	2	
▪ No abnormal findings	2	2	
Inability to puncture the port	2 (7.7%)	4 (10.0%)	0.56
▪ Irreversible port reversal	1	1	
▪ No abnormal findings	1	3	
Swelling of the neck/arm	2 (7.7%)	2 (5%)	0.52
▪ Venous occlusion	1	0	
▪ No abnormal findings	1	2	
Pain of the neck/arm	1 (3.8%)	2 (5.0%)	0.66
▪ Venous occlusion	1	0	
▪ Catheter-related injury	0	1	
▪ Catheter malposition into IJV	0	1	
Reason for CVP removal (*N*)	5 (19.2%)	12 (30.0%)	0.25
▪ Fibrin sheath formation	1	1	
▪ Permanent obstruction	1	3	
▪ Irreversible port reversal	1	1	
▪ Venous occlusion	2	0	
▪ Permanent catheter kinking	0	3	
▪ Catheter-related injury	0	3	
▪ Catheter malposition into IJV	0	1	

*CVP* Central venous port, *IJV* Internal jugular vein

The CVP was removed in 8/390 and 13/800 patients in the arm and chest wall groups, respectively, because of infection (catheter infection and port pocket infection) or because FCS seemed inappropriate. The incidence of CVP-related infection was 1.6/100 and 1.3/100 catheter days in the arm and chest wall groups, respectively.

## Discussion

FCS was effective in deciding whether to continue using CVP and prevent unnecessary CVP removal. In addition, the interval between CVP implantation and FCS in the upper arm group was shorter than that in the chest wall group. Most reasons for dysfunction were temporary obstruction; hence, it was possible to continue using the CVP.

In a previous report, “pinch-off syndrome” occurred in approximately 1% of subclavian procedures [7]. However, catheter-related injury did not occur in the upper arm group because of anatomical conditions. Distal catheter migration from the puncture point is also unlikely in straightforward upper arm central vein lines because of the absence of sharp turns causing tension in an elastic restoring force. Such turns are usually encountered during subclavian or internal jugular procedures [6]. Hence, we switched from subclavian implantation to upper arm CVP implantation in our institution.

In our institution, pneumothorax occurred in 2% of the CVPs implanted in the subclavian vein; however, the CVPs were successfully placed in the arms of all patients without any implantation-related complications, including pneumothorax, hemothorax, or cardiovascular problems.

Considering its safety, the use of indwelling arm CVPs is expected to increase in the future. However, there is no consensus on the management of mechanical complications in the arm. In general, the most common complaints are prolonged infusion time and inability or resistance to saline injection during CVP [2]. These complaints are often because of catheter kinking, catheter occlusion, and fibrin sheath formation [2]. In our study, the main complaints and mechanical complications were similar in both the upper arm and chest wall groups.

In the chest wall group, the incidence of unnecessary removal/reimplantation of the CVP after FCS was similar to that in a previous report [2]. There have been no reports on the FCS results of CVP in the upper arm in the past. Furthermore, no previous study has examined the differences in mechanical complications between the upper arm and chest wall CVP. In our study, temporary obstruction occurred more frequently in the upper arm group. Occlusion seems more likely in the upper arm group because of the longer indwelling catheter; hence, increasing the amount of flash and pulsative flushing after use may be necessary in the upper arm group.

Even after uneventful implantation, proper catheter maintenance is essential to avoid mechanical complications. Knowledge regarding the possible mechanical complications is a prerequisite to avoid unnecessary removal or reimplantation.

Our study has certain limitations. Our study was a retrospective study with a small sample size. A randomized clinical trial comparing the safety and utility of upper arm and chest wall CVPs (including internal jugular vein access) is warranted. Because performing FCS and deciding whether CVP can still be used were based on the physician’s discretion, a bias in patient selection may be present.

## Conclusion

FCS was effective in evaluating CVP system-related mechanical complications in both the upper arm and chest wall groups. FCS was also useful in deciding whether CVP system removal and reimplantation were required. The duration of CVP implantation until the FCS was shorter in the upper arm group compared to the chest wall group. However, because most mechanical complications were temporary obstructions, it was possible to continue using the CVP.

## Data Availability

The datasets generated and/or analyzed during the current study are not publicly available due to patients’ privacy but are available from the corresponding author on reasonable request.

## Abbreviations

CVP: Central venous port
FCS: Flow confirmation study
US: Ultrasonography
DSA: Digital subtraction angiography
